# Partial Nephrectomy Versus Radical Nephrectomy for Clinical T2 or Higher Stage Renal Tumors: A Systematic Review and Meta-Analysis

**DOI:** 10.3389/fonc.2021.680842

**Published:** 2021-06-10

**Authors:** Ruizhen Huang, Chiyu Zhang, Xing Wang, Honglin Hu

**Affiliations:** Department of Urology, The Second Affiliated Hospital of Nanchang University, Nanchang, China

**Keywords:** kidney cancer, partial nephrectomy, radical nephrectomy, meta-analysis, renal function, oncologic outcome

## Abstract

**Objective:**

The choice of surgical method for clinically diagnosed T2 or higher stage kidney cancer remains controversial. Here, we systematically reviewed and collected published comparative studies on renal function, oncologic outcomes, and perioperative results of partial nephrectomy (PN) versus radical nephrectomy (RN) for larger renal tumors (T2 and above), and performed a meta-analysis.

**Evidence Acquisition:**

Following searches of PubMed, Web of Science, and Embase, the original studies on PN vs. RN in the treatment of T2 renal cancer were screened through strict inclusion and exclusion criteria. RevMan 5.4 was used for data analysis of the perioperative results, renal function, and oncologic outcomes of the two surgical methods for T2 renal tumor therapy. The weighted mean difference was used as the combined effect size for continuous variables, while the odds ratio (OR) or risk ratio (RR) was used as the combined effect size for binary variables. Both variables used a 95% confidence interval (CI) to estimate statistical accuracy. In cases with low heterogeneity, the fixed-effects model was used to pool the estimated value; otherwise, the random-effects model was used when significant heterogeneity was detected.

**Results:**

Fifteen retrospective studies including 5,056 patients who underwent nephrectomy (PN: 1975, RN: 3081) were included. The decline in estimated GFR (eGFR) after PN was lower than RN [(MD: −11.74 ml/min/1.73 m^2^; 95% CI: −13.15, −10.32; p < 0.00001)]. The postoperative complication rate of PN was higher than that of PN (OR: 2.09; 95% CI: 1.56, 2.80; p < 0.00001)], and the postoperative overall survival (OS) of PN was higher than that of RN (HR: 0.77; 95% CI: 0.65, 0.90; p = 0.002), and tumor recurrence (RR, 0.69; 95% CI: 0.53, 0.90; p = 0.007). No obvious publication bias was found in the funnel chart of the OS rates of the two groups of patients.

**Conclusions:**

PN is beneficial for patients with T2 renal tumors in terms of OS and renal function protection. However, it is also associated with a higher risk of surgical complications.

## Introduction

Kidney cancer is a common tumor in the urinary system. According to the EAU Urology Kidney Cancer Diagnosis and Treatment Guidelines updated in 2014, radical nephrectomy (RN) should be performed for renal tumors of clinical T staging T2, or in patients with localized renal tumors that cannot be treated with nephron preservation (1). Clinically, for patients with localized T1a-b renal tumors, both partial nephrectomy (PN) and RN have been shown to have similar oncological effects. At the same time, studies have shown that patients with PN have less postoperative renal function decline than RN. Therefore, regardless of whether artificially-assisted laparoscopic or robot-assisted laparoscopic PN, PN is regarded as the best choice for the treatment of T1 renal tumors. It has been reported that the 10-year overall survival (OS) rate of patients with T1 stage renal tumors who underwent laparoscopic or open partial nephrectomy was associated with the patient’s age, comorbidities, surgical indications, and other factors, as well as the prediction of cancer-free survival rate, but not the surgical method itself. The choice of surgical method depends only on the surgeon’s preference and experience (2).

RN has long been the preferred surgical method for the treatment of T2 stage and larger renal tumors. Unfortunately, although this surgical method can effectively remove the tumor, the higher risk of postoperative renal insufficiency, postoperative bleeding, infection, and other underlying complications remain problematic compared to PN (3–5). Several observational studies have reported improved survival benefits for patients undergoing PN compared to those undergoing RN. The choice of PN over RN is considered important from a therapeutic perspective, because PN is associated with improved renal functional preservation, and may therefore be beneficial for prognosis and OS by lowering the risk of cardiovascular and metabolic sequelae (4, 6–8). Taken together, it seems, to some extent, that PN is superior to RN in the surgical management of renal cancer. However, recent studies involving PN in stage T2 or higher kidney tumors have attracted widespread attention, and among patients with larger kidney masses, PN does not compromise cancer-specific mortality. However, the choice of surgical method remains controversial. Therefore, we performed a systematic literature review and meta-analysis to summarize the efficacy and safety of PN vs. RN in the treatment of stage T2 or higher renal tumors.

## Methods

The literature filter approach, search methods, information collection procedure, measured outcomes, and results synthesis were defined prospectively adhering to the PRISMA guidelines (9).

### Criteria for Considering Studies

Original articles that met the following criteria were considered for inclusion: Original articles that involved studying the efficacy of PN vs. RN in the treatment of T2 renal tumors, or some subgroups; publications written in the English language; studies in which the patients undergoing nephrectomy were all adults (> 18 years old); both retrospective and prospective studies; studies meeting at least one of the required outcome indicators for this study. The exclusion criteria were as follows: Non-PN vs. RN research; comments, conference abstracts, reviews, or replies, in which relevant data could not be extracted; case reports; and studies in which the research subjects were animals, cells, or minors. The abstracts of the studies that met the inclusion criteria were scanned, and for those that met the requirements, a more comprehensive evaluation was performed by reviewing the full text. We also reviewed the reference lists of the original documents, and, to avoid unnecessary omission of any original documents required, we checked the references of similar meta-analysis articles.

### Search Methods

**Figure 1:**
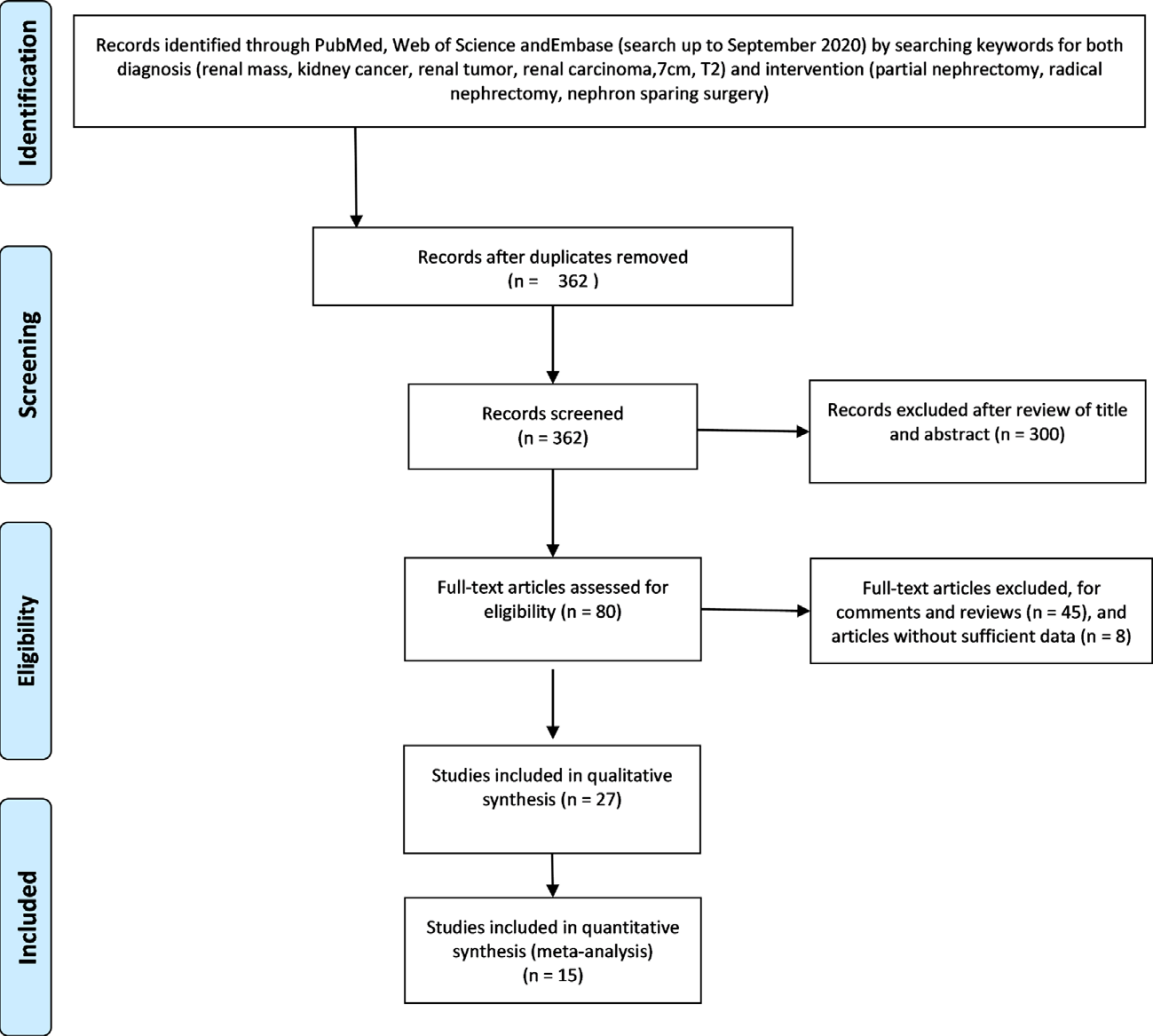
PRISMA flow diagram of the search strategy and identification of relevant studies (10).

We performed a search of the original research from PubMed database, Web of Science, and Embase from inception until September 2020. The search terms included the diagnostic terms “kidney mass” OR “renal cancer” OR “renal tumor” OR “7 cm” or “T2,” and the treatment intervention items “partial nephrectomy” OR “radical nephrectomy” OR “nephron-sparing surgery”. All steps were based on the meta-analysis of the PRISMA statement flow chart (**Figure 1**), and a comprehensive evaluation and data extraction was performed for the remaining articles.

### Data Extraction

Prior to data extraction, an Excel table was prepared to store the required data. The data were extracted by RH and CZ in strict accordance with the inclusion and exclusion criteria, respectively. Any questions or disagreements regarding the data extraction from the two authors were negotiated with a third author (HH) to determine the final extraction plan. According to the collected statistics, the extracted information included literature characteristics (including author, case sources, study type, study period, number of cases in the PN and RN groups, surgical methods, average follow-up time), demographic characteristics (age, sex, tumor size, baseline renal function), perioperative outcomes (operation time, estimated blood loss, complications, length of stay in hospital), renal function outcomes (postoperative estimated GFR [eGFR], decline in eGFR), and tumor outcomes (tumor recurrence rate, overall survival rate, cancer-specific mortality, cancer-specific survival, and all-cause mortality).

### Quality Assessment

The quality of the literature to be included was independently evaluated by RH and CZ. A third researcher (XW) independently evaluated the quality of the literature if there were divergences between the former two researchers. The results were then scored and a final decision was made. For randomized controlled trials, we used the Cochrane Systematic Evaluation Manual to conduct a comprehensive risk of bias assessment, including selection bias, outcome bias, test bias, attrition bias, and publication bias. The Newcastle-Ottawa scale (11) Documents with a score of ≤ 5 were considered low-quality, a score of 6–7 were classified as medium quality, and those with a score of 8–9 were classified as high-quality. The standards implemented by the Oxford University Center for Evidence-Based Medicine were used to further assess the level of documentary evidence (12).

### Statistical Processing

RevMan 5.4, as recommended by the Cochrane manual, was used to statistically analyze the outcome indicators of each of the included studies. The weighted mean difference (WMD) was used as the combined effect size for continuous variables, while the odds ratio (OR) or risk ratio (RR) was used as the combined effect size for binary variables. Both variables used a 95% confidence interval (CI) to estimate statistical accuracy. The heterogeneity between each study was evaluated using Chi-square and I^2^, and statistical significance was set at p < 0.10. The random-effects model was used to combine the effect size in outcomes with significant heterogeneity (p < 0.1, I^2^ > 50%); otherwise, the fixed effects model was used (13). Sensitivity analysis was tested by the literature elimination method, in which the literature with the lowest weight and the literature with the lowest quality score in each index were eliminated to test whether the main results were robust. Publication bias was evaluated by visually inspecting the funnel plot.

Original literature in which data were presented as the median and interquartile ranges. Referred the validated mathematical model of (14, 15) to calculate the index data of the original literature and acquire the mean and standard deviation. The data conversion method was used to extract outcome indicators, such as cancer-specific survival (CSS), a natural logarithmic transformation of the HR of the original document [e.g., ln (HR)], thereby transforming the converted values into forest plots.

## Results

Fifteen retrospective studies including 5,056 patients who underwent nephrectomy (PN, 1975; RN, 3081) were included by careful selection (16–30). Among the included studies, the majority of cases were from the USA, and the remainder were varied. Single-center studies accounted for 60%, and the remaining 40% were multicenter studies. Except for some documents that did not clearly state the surgical method, most open surgeries were included in the documents. Interestingly, two original types of research on minimally invasive surgery are robot-assisted surgery (24, 27). The characteristics of the included studies are summarized in **Table 1**.

**Table 1:** Characteristics of the included studies.

Reference	Study origin	Design	Study period	Surgical methods	PN/RN		NOS score	Evidencelevel
					Cases (n)	Mean FU (M)		
(16)	Canada, France	RTP, MI	1984–2001	Unspecified	17/45	57.6/55.2	8	III
(17)	USA	RTP, MI	2002–2012	Open/lap	80/122	41.5/41.5	8	II
(18)	USA	RTP, PM, SC	1970–2008	Unspecified	69/207	38.4/38.4	8	III
(19)	USA	RTP, SC	1990–2006	Unspecified	34/567	62.1/43.4	7	III
(20)	Germany	RTP, SC	1988–2007	Open	16/28	56.4/45.6	6	III
(21)	France	RTP, SC	2000–2013	Open/lap	49/81	31/45	8	III
(29)	Maryland	RTP, SC	2003–2015	Open/lap	437/350	32.9/38.7	7	III
(22)	USA	RTP, SC	2004–2010	Open	45/108	Unknown	6	III
(23)	Germany	RTP, MI	1980–2010	Open/lap	18/105	163/93	6	II
(24)	USA	RTP, SC	2000–2012	Open/lap/robot	66/231	Unknown	5	III
(25)	Israel	RTP, SC	2012–2017	Lap	13/16	44.5/44.5	7	III
(30)	Multi-national	RTP, MI	1992–2003	Unspecified	268/273	111.6/111.6	5	III
(26)	USA	RTP, SC	1988–2008	Unspecified	245/245	60/60	7	III
(27)	France	RTP, MI	2000–2014	Open/lap/robot	91/176	24/24	7	III
(28)	USA	RTP, MI	2004–2009	Unspecified	527/527	49.2/49.2	6	III

RTP, Retrospective; MI, Multi-institutional; SC, Single-center; Lap, Laparoscope; FU, Follow-up; M, Month.

**Figure 2:**
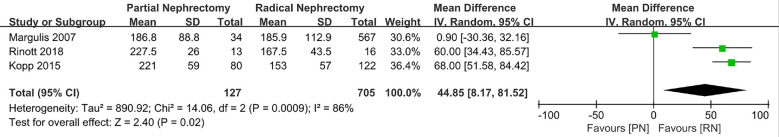
Operative time.

**Figure 3:**
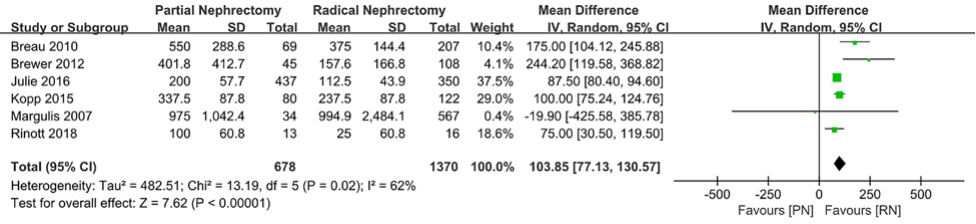
Estimated blood loss.

**Figure 4:**
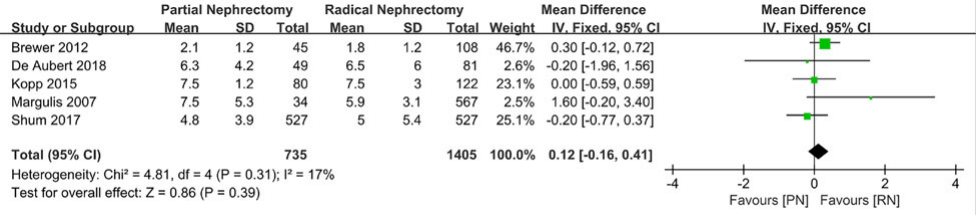
Length of stay.

Statistical analysis of some perioperative indicators (operating time, hospitalization time, and estimated blood loss during surgery) showed that compared to RN, patients who underwent PN had a longer operation time than those who underwent RN (MD: 44.85 min, 95% CI: 8.17, 81.52, p = 0.02; **Figure 2**), as well as a higher likelihood of estimated blood loss (MD: 103.85 ml, 95% CI: 77.13, 103.57; p < 0.00001, **Figure 3**). As for the length of stay, there was no significant difference between PN and RN. We combined the data of the length of stay in the literature and found no significant difference (MD: 0.12 days; 95% CI: **−**0.16, 0.41; p = 0.39, **Figure 4**).

**Figure 5:**
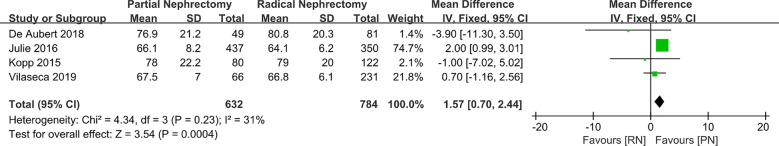
Preoperative eGFR.

**Figure 6:**
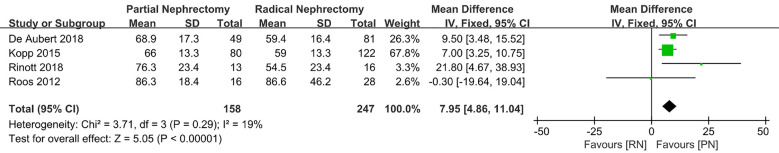
Postoperative eGFR.

**Figure 7:**
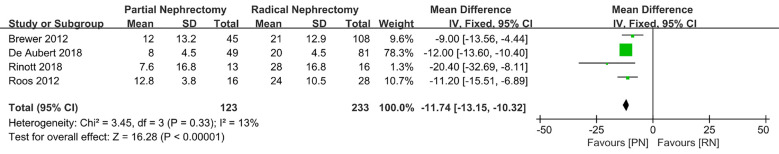
Decline in eGFR.

**Figure 8:**
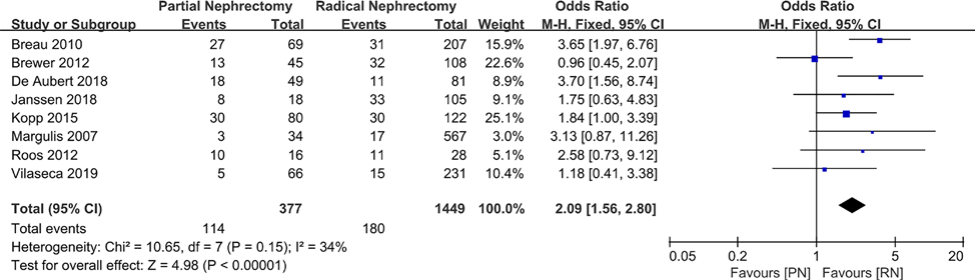
Complications.

We used the estimated glomerular filtration rate (eGFR) to estimate renal function and compared the preoperative eGFR, postoperative eGFR, and the decrease in eGFR (ΔeGFR: preoperative eGFR minus postoperative eGFR). The pooled effect size indicated that the preoperative eGFR was higher in patients with PN, and that these patients also had better baseline renal function (MD: 1.57 ml/min/1.73 Â m^2^; 95% CI: 0.70, 2.44; p = 0.0004; **Figure 5**). Moreover, after a short follow-up time, the postoperative renal function of patients who underwent RN was slightly worse than that of patients who underwent PN, as represented by the higher postoperative eGFR in the PN group (MD: 7.95 ml/min/1.73 Â m^2^; 95% CI: 4.86, 11.04; p < 0.00001; **Figure 6**). The renal function of the patients in both groups decreased following surgery. Our combined data analysis showed that ΔeGFR was statistically significant in the PN and RN groups, and that the decline in eGFR was even lower in the PN patients (MD: **−**11.74 Â min/1.73 m^2^; 95% CI: **−**13.15, **−**10.32; p < 0.00001; **Figure 7**); these findings indicated significantly better preservation of renal function in the PN group. Furthermore, the pooled results suggest a tendency for a lower complication rate in patients after RN surgery than after PN (OR: 2.09; 95% CI: 1.56, 2.80; p < 0.00001, **Figure 8**); thus, it seems that RN is superior in controlling postoperative complications.

**Figure 9:**
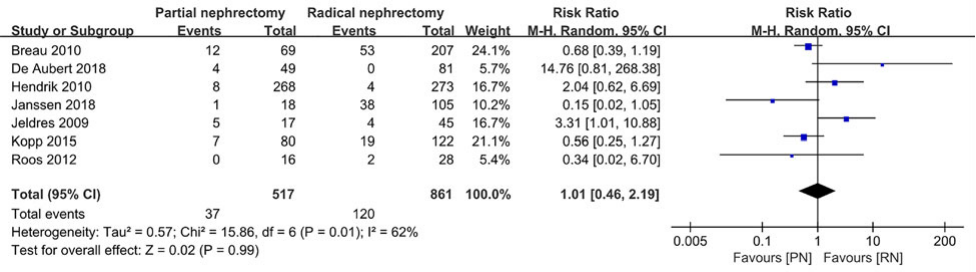
Cancer-specific mortality.

**Figure 10:**
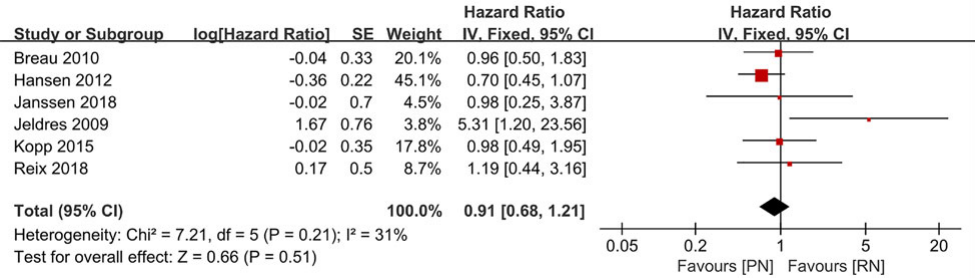
Cancer-specific survival.

**Figure 11:**
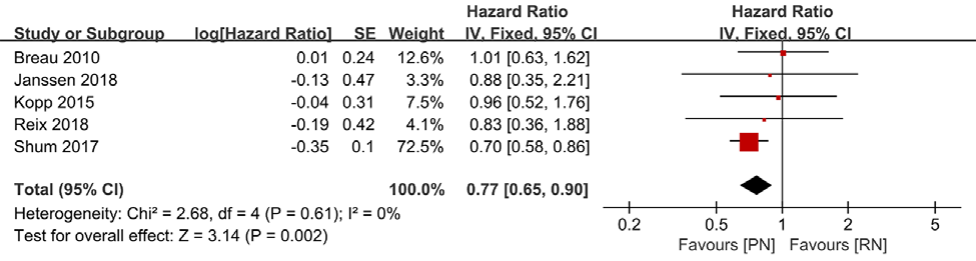
Overall survival.

**Figure 12:**
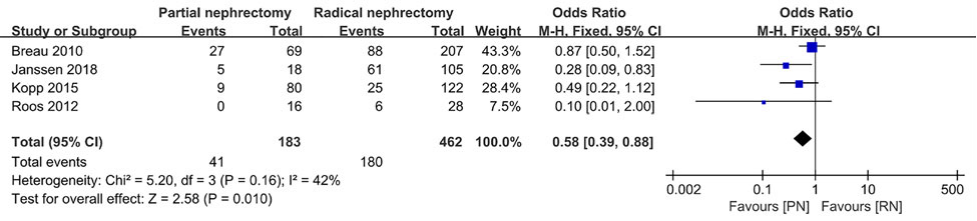
All-cause mortality.

**Figure 13:**
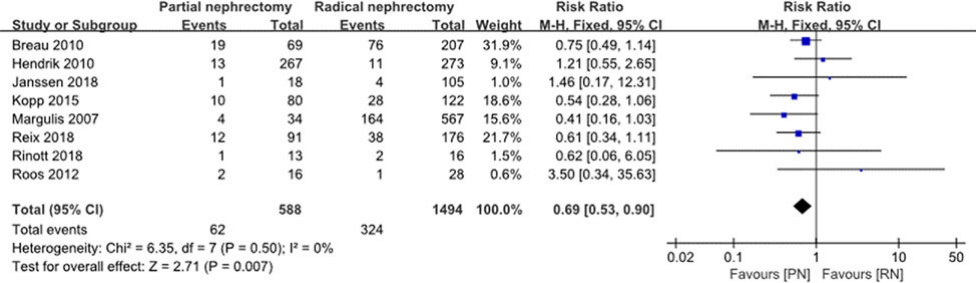
Tumor recurrence.

With regard to the long-term outcome indicators, we failed to find any obvious differences between pooled cancer-specific mortality and cancer-specific survival, which indicate that PN and RN are not significantly different in the above two aspects (combined results, RR: 1.01; 95% CI: 0.46, 2.19; p = 0.99; **Figure 9**; and HR: 0.91; 95% CI: 0.68, 1.21; p = 0.66; **Figure 10**). The combined results showed a clear difference between PN and RN in terms of OS (HR: 0.77; 95% CI: 0.65, 0.90; p = 0.002; **Figure 11**). Patients who underwent PN generally had a longer OS than those who underwent RN, with low heterogeneity (I^2^ = 0%) among the included literature, showing a relatively stable pooled result. The combined effect size of all-cause mortality of PN patients was lower than that of RN patients (OR: 0.58; 95% CI: 0.39, 0.88; p = 0.01, **Figure 12**), also was it in the pooled tumor recurrence indicators (RR: 0.69; 95% CI: 0.53, 0.90; p = 0.007, **Figure 13**).

**Figure 14:**
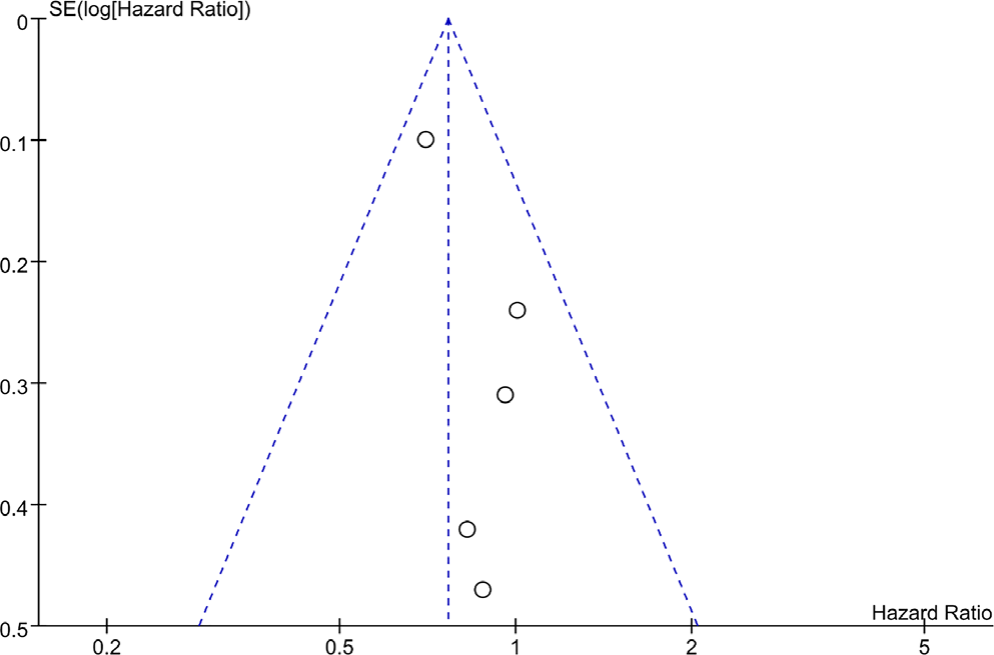
Funnel plot concerning overall survival.

Sensitivity analysis of each combined result was performed by eliminating the original documents with the lowest weight and the lowest quality score in each outcome index one by one. As a result, the changes in p-values and the heterogeneity of the combined indicators were recorded and composed in the form of a table. Most of the outcome indicators did not change significantly after removing the relevant original literature one by one. By removing the lowest weighted reference study of the operation time, the heterogeneity was significantly changed from 86% to 0%, but after excluding the lowest quality score, there was no significant change (**Table 2**). In this study, the random-effects model was used to combine the effect size for the indicators with high heterogeneity (I^2^ > 50%); otherwise, the fixed effects model was used. In order to detect related publication bias, a funnel chart of OS was reported, as shown in **Figure 14**. All included documents were within 95% CI, and no obvious publication bias was observed.

**Table 2:** Results of sensitivity analysis about comparison of PN vs. RN.

Inclusion results	Studies (n)	Patients (PN, n)	Patients (RN, n)	Effect measure (95%CI)	P-value	Heterogeneity			
						Chi^2^	df	P	I^2^ (%)
**Perioperative outcomes**									
**Length of stay**	5	735	1405	MD 0.12 (−0.16, 0.41)	0.39	4.81	4	0.31	17
Exclusion of the lowest weight (19)	4	701	838	MD 0.09 (−0.20, 0.38)	0.56	2.16	3	0.54	0
Exclusion of the lowest score (21)	4	686	1324	MD 0.13 (−0.26, 0.42)	0.37	4.68	3	0.2	36
**Operative time**	3	127	705	MD 44.85 (8.17, 81.52)	0.02	14.06	2	0.00009	86
Exclusion of the lowest weight (19)	2	93	138	MD 65.67 (51.85, 79.48)	0.00001	0.27	1	0.61	0
Exclusion of the lowest score (25)	2	114	689	MD 35.82 (−29.88, 101.52)	0.29	13.87	1	0.0002	93
**Estimated blood loss**	6	678	1370	MD 103.85 (77.13, 130.57)	0.00001	13.19	5	0.02	62
Exclusion of the lowest weight (19)	5	644	803	MD 105.02 (77.4, 132.64)	0.00001	12.91	4	0.01	69
Exclusion of the lowest score (25)	5	665	1354	MD 113.61 (80.41, 146.8)	0.00001	12.78	4	0.01	69
**Renal function outcomes**									
**Preoperative eGFR**	4	632	784	MD 1.57 (0.7, 2.44)	0.0004	4.34	3	0.23	31
Exclusion of the lowest weight (21)	3	583	703	MD 1.65 (0.77, 2.53)	0.0002	2.21	2	0.33	9
Exclusion of the lowest score (24)	3	566	553	MD 1.82 (0.83, 2.80)	0.0003	3.26	2	0.20	39
**Postoperative eGFR**	4	158	247	MD 7.95 (4.86, 11.04)	0.00001	3.71	3	0.29	19
Exclusion of the lowest weight (20)	3	142	219	MD 8.17 (5.04, 11.30)	0.00001	2.99	2	0.22	33
Exclusion of the lowest score (25)	3	145	231	MD 7.49 (4.35, 10.63)	0.00001	1.12	2	0.57	0
**Decline in eGFR**	4	123	233	MD −11.74 (−13.15, −10.32)	0.00001	3.45	3	0.33	13
Exclusion of the lowest weight (25)	3	110	217	MD −11.62 (−13.04, −10.20)	0.00001	1.52	2	0.47	0
Exclusion of the lowest score (25)	3	110	217	MD −11.62 (−13.04, −10.20)	0.00001	1.52	2	0.47	0
**Oncologic outcomes**									
**Overall survival**	5	785	1137	HR 0.77 (0.65, 0.9)	0.002	2.68	4	0.61	0
Exclusion of the lowest weight (23)	4	767	1032	HR 0.76 (0.64, 0.9)	0.002	2.59	3	0.46	0
Exclusion of the lowest score (23)	4	767	1032	HR 0.76 (0.64, 0.9)	0.002	2.59	3	0.46	0
**Cancer-specific survival**	6	520	900	HR 0.91 (0.68, 1.21)	0.51	7.21	5	0.21	31
Exclusion of the lowest weight (16)	5	503	855	HR 0.85 (0.63, 1.14)	0.27	1.59	4	0.81	0
Exclusion of the lowest score (26)	5	275	655	HR 1.13 (0.76, 1.67)	0.55	4.6	4	0.33	13
**Cancer-specific mortality**	5	451	735	RR 0.63 (0.42, 0.95)	0.03	6.13	4	0.19	35
Exclusion of the lowest weight (20)	4	435	707	RR 0.64 (0.42, 0.97)	0.03	5.91	3	0.12	49
Exclusion of the lowest score (23)	4	433	630	RR 0.74 (0.49, 1.13)	0.16	3.57	3	0.31	16
**Long-term indicators**									
**Complications**	8	377	1449	OR 2.09 (1.56, 2.80)	0.00001	10.65	7	0.15	34
Exclusion of the lowest weight (19)	7	343	882	OR 2.06 (1.53, 2.78)	0.00001	10.28	6	0.11	42
Exclusion of the lowest score (24)	7	311	1218	OR 2.20 (1.62, 2.98)	0.00001	9.32	6	0.16	36
**Recurrence**	8	588	1494	RR 0.69 (0.53, 0.9)	0.007	6.35	7	0.5	0
Exclusion of the lowest weight (20)	7	572	1466	RR 0.67 (0.52, 0.88)	0.004	4.51	6	0.61	0
Exclusion of the lowest score (25)	7	575	1478	RR 0.69 (0.53, 0.91)	0.007	6.34	6	0.39	5
**All-cause mortality**	4	183	462	RR 0.70 (0.53, 0.93)	0.01	5.35	3	0.15	44
Exclusion of the lowest weight (20)	3	167	434	RR 0.73 (0.55, 0.97)	0.03	3.63	2	0.16	45
Exclusion of the lowest score (23)	3	165	357	RR 0.76 (0.56, 1.03)	0.07	3.59	2	0.17	44

**Figure 15:**
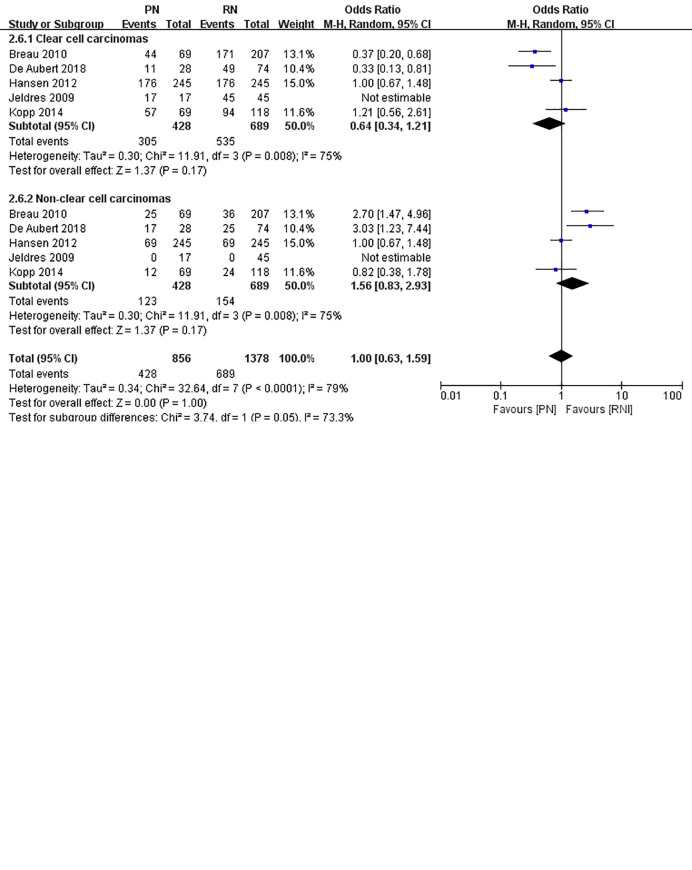
Subgroup analysis of Fuhrman Grade and cancer histology.

We categorized the trials into two subgroups: clear cell carcinoma group and non-clear cell carcinoma group according to the cancer histology; based on the Fuhrman Grade, the low-grade (I-II) group and the high-grade (III-IV) group were also considered to be analyzed. Accordingly, 5 trials were included in the cancer histology subgroup, and 5 trials were also included in the Fuhrman Grade subgroup (**Figure 15**). The OR value in the low-grade subgroup was 1.13 (95%CI, 0.86, 1.49) and that in the high-grade subgroup was 0.84 (95% CI; 0.64, 1.01). Furthermore, the OR value in the clear cell carcinoma group was 0.64(95%CI, 0.34, 1.21) and that in the and non-clear cell carcinoma was 1.56 (95% CI; 0.83, 2.93). However, a high degree of heterogeneity was observed in the cancer histology subgroup (I^2^ = 75%).

## Discussion

With advances in science and technology, PN for the treatment of larger kidney tumors is no longer theoretically feasible. However, RN is still regarded as a surgical method with greater reference value for larger clinical T2 renal tumors ≥ 7 cm. Based on this, the specific implementation of PN or RN remains controversial. The purpose of this study was to conduct a detailed systematic review and meta-analysis to evaluate whether the clinical diagnosis of T2 renal tumors should be PN or RN. To better understand the advantages and disadvantages of PN and RN in the treatment of T2 renal tumors, statistical analysis was performed with the aim to provide a reference for controversy in choosing PN or RN in cancer therapy and citing published retrospective research sample data for combined statistical analysis. We found that PN required a longer operation time (MD: 44.85 min; 95% CI: 8.17, 81.52; p = 0.02) and had higher intraoperative blood loss than RN (MD: 103.85 ml; 95% CI: 77.13, 103.57; p < 0.00001), which is a more technically demanding surgical method. PN also results in more complications compared to RN. Despite the above advantages, the benefits of RN in terms of OS and renal function protection are minimal compared to those of PN in the treatment of T2 stage renal cancer.

Because of a similar oncology control effect as RN, PN is also efficient in retaining residual renal function and reducing the incidence of postoperative chronic kidney disease in patients, which further benefits the cardiovascular system and the OS of patients. For T2 stage renal tumors, our combined effect size showed that neither PN nor RN was statistically significant in terms of cancer-specific mortality and cancer-specific survival rates. These findings indicate similarities in the effects of oncologic control in T2 stage renal tumors. Alanee et al. (31) obtained data from the SEER database to analyze the results of the surgical treatment of T2 renal tumors, and showed that compared to RN-treated patients, the cancer-specific mortality rate of PN was not inferior to RN (HR: 0.68; 95% CI: 0.50 –0.94). Further investigation showed that sex, age, race, tumor size > 10 cm, localized disease, and histopathological classification were all related to improved survival rate following PN (all p-values < 0.05). However, Hansen et al. (26) suggested that even in patients with relatively unfavorable tumor pathological results, PN would not reduce cancer-specific mortality, which was supported by the results of their multivariate analysis (HR, 0.67; 95% CI, 0.39–1.17, p = 0.2). Furthermore, according to research by Kopp et al. (17, 32), patients with renal cancer with R.E.N.A.L. scores ≥ 10 at stage T2 had an increased risk of tumor progression and decreased OS, suggesting that R.E.N.A.L. scores may be a more accurate reference tool for evaluating tumor prognosis than simple pathological staging. In our combined results, OS (HR: 0.77; 95% CI: 0.65, 0.90; p = 0.002), one of the main efficacy evaluation indexes of cancer treatment, showed significant differences between the two therapies, and the OS of the PN group was significantly better than that of the RN group. As the average follow-up time of each retrospective study that met the inclusion criteria was generally different (PN ranges from 24–163 months, RN ranges from 24–111.6 months), loss to follow-up deviation may still exist. Therefore, even if the statistics are not heterogeneous after merging, they still pose a huge information deviation. A larger sample in a multi-center, multi-country, multi-ethnic comprehensive analysis is essential. However, the combined OS in this study is still meaningful and can be used as a reference to provide a basis for clinical treatment.

For larger renal cell carcinoma, both the difficulty of the surgical procedure and the incidence of postoperative complications are increased while selecting partial nephrectomy as the strategy. A retrospective study conducted by Kopp et al. (17, 32) in the same patients population examined two different indicators: the survival rate and renal function score of PN vs. RN. They revealed that the two surgical methods had no significant difference in terms of the renal function score and survival rate of patients with T2 tumors, but that the incidence of complications in the PN group was significantly higher than that in the RN group (17.5% vs. 2.5%; p < 0.001), and 10% of patients had urine leakage after surgery. Furthermore, the operative time of patients who underwent PN was significantly longer than that of those who underwent RN (221 min vs. 153 min, p = 0.001). A longer operation time means longer intraoperative exposure, longer conditions of stress and ischemia, and longer tumor resection time and kidney reconstruction time than RN, all of which may lead to an increased incidence of postoperative complications. In addition, the larger the tumor, the larger the scope of resection, and the more complex the nature of the tumor, the higher the technological requirements for the surgeon. A larger resection scope can reduce the benefits of renal function, which are important risk factors the incidence of complications. During a prospective, randomized study in nephron sparing surgery (NSS)or RN conducted by the European Organization for Research and Treatment of Cancer Genito-Urinary Group (EORTC-GU) noninferiority phase 3 trial 30904, approximately 50% of the study subjects had T2 stage renal cancer. The results of the study found that in PN vs. RN, the incidence of bleeding (3.1% vs. 1.2%), urinary fistula (4.4% vs. 0%), and the rate of secondary operations (4.4% vs. 2.4%) were higher in the PN group (33). Studies have reported (34) that PN was associated with a significant incidence of complications and reduced renal function in patients with renal cancer > 7 cm. However, the postoperative results of selective indication PN were surprising in that they were similar to those of RN. In the case of selecting indications, the estimated values of 5-year cancer-free survival (CFS), CSS, and OS rates were 85.7%, 98%, and 93.9%, respectively, which were significantly higher than the 5-year CFS (60.5%), CSS (78.5%), and OS (70.6%) in the imperative indication group. In the sample study population, the necessary indications [defined as bilateral tumors, preoperative kidney disease (CKD stage < 2), and solitary kidney] and selective indications seem to play a key role in the prognosis. Breau et al. (18) retrospectively studied PN and RN and reported a low complication rate in both groups, and that urine leak in the PN group was resolved spontaneously under the action of the drainage tube and/or when the ureteral stent placed after the abdominal cavity was closed. Their research provides strong evidence for the safety and effectiveness of PN in the treatment of T2 renal cancer. In terms of postoperative complications, the risk of PN treatment for T2 renal cancer is also acceptable. Schwentner et al. (23) performed a study with a mean follow-up time of up to 102 months and confirmed that it is feasible to implement PN for renal cancer ≥ 7 cm in terms of acceptable technology and complications. Our combined results showed a lower tumor recurrence rate in the PN group (RR: 0.69; 95% CI: 0.53, 0.90; p = 0.007). When performing PN, patients are at a higher risk of local recurrence; therefore, the patients with more aggressive disease were probably managed with RN, resulting in a higher recurrence rate. As RN tends to be the preferred choice of surgery for larger tumors, selection bias may have led to this combined result, and a larger sample size investigation should be conducted for further validation. Based on these studies and our combined effect on complications, we believe that nephron-sparing surgery should be considered in patients with stage T2 RCC. The strict control of surgical indications and tumor conditions before PN surgery will provide the greatest benefits to patients.

Preoperative eGFR (17, 21, 24, 29) in the original literature, postoperative eGFR (17, 20, 21, 25), and decline in eGFR (20–22, 25) within a limited follow-up period to evaluate the effect of PN vs. RN on renal function. Cumulative analysis of the included studies showed that the preoperative eGFR was higher and the baseline renal function was better in patients who underwent PN (MD: 1.57 ml/min/1.73 Â m^2^; 95% CI: 0.70, 2.44; p = 0.0004). In addition, the postoperative eGFR in the PN group was higher than that in the RN group (MD: 7.95 Â ml/min/1.73 Â m^2^; 95% CI: 4.86, 11.04; p < 0.00001). ΔeGFR was significant in both PN and RN, but showed a smaller reduction in PN (MD: –11.74 Â ml/min/1.73 m^2^; 95% CI: –13.15, –10.32; p < 0.00001), indicating that PN is superior to RN in terms of preserving renal function. Due to the advantages of PN in preserving nephrons and protecting renal function, the use of PN for the treatment of larger renal tumors is justified. Clark et al. (3) dynamically measured the changes in 24-h urine CrCl and used the Cockcroft–Gault formula to calculate the eGFR. Their results demonstrated that the effect of PN on the deterioration of renal function during the postoperative period was minimal, while the effect of RN was pronounced. According to the results of Kopp et al. (17, 32), the average eGFR decreased more in patients with T2 renal cancer who underwent RN than those who underwent PN (–19.7 vs. –11.9 ml/min; p = 0.006); at the same time, the incidence of CKD caused by decreased renal function increased (40.2% vs. 16.3%; p < 0.001). Low-level eGFR accompanied by renal insufficiency may be related to the risk of increased mortality, increased cardiovascular events, and prolonged hospital stay. In line with this, the lower the eGFR value classification of CKD at each stage, the higher the risk of complications (8).

The results of comparative analysis showed 1121 patients with incident stage 4 or higher CKD, including 183 patients who underwent PN and 938 patients who underwent RN. Patients with larger tumors (T2) treated with PN showed an increased incidence of clinically significant postoperative CKD than those with T1 tumors (35). Besides, Mariusdottir et al. (36) revealed that the development of new-onset CKD was significantly lower after PN than after RN [n = 9 (*20%) vs. 19 (43%), p = 0.002]. Multivariate logistic regression analysis indicated that RN was an independent prognostic factor for new-onset CKD (OR = 3.07; 95% CI, 1.03–9.79; p = 0.04), which indicates that the renal function of patients with renal cancer should be assessed before surgery. If the patient’s CKD stage reaches stage 2, especially stage 2b, when PN is feasible, the strategy of nephron preservation surgery should be considered (37). A similar study was conducted by Chung et al. (38), the results of which demonstrated that compared to RN, PN had a more favorable effect on the OS of patients with renal dysfunction (CKD stage I and II) before surgery. However, for patients with CKD stage III, PN showed no obvious improvement in postoperative renal function, with no significant improvement in 5-year mortality.

To the best of our knowledge, the operation time of PN is longer than that of RN, irrespective of whether it is an open or minimally invasive surgery. According to the statistics of our hospital database, PN has a longer intraoperative time, even in patients with relatively small renal tumors. Additionally, previous literature shows that the average operation time of PN vs. RN in patients with T2 or greater renal tumors can be referred to. Our combined results showed the same result (MD: 44.85 min; 95% CI: 8.17, 81.52; p = 0.02). It is also worth noting that the combined effect size was heterogeneous (I^2^ = 86%). In the sensitivity analysis, we removed the lowest weighted reference (19) and found that the I^2^ dropped to 0%, while the literature with the lowest quality score (25) remained highly heterogeneous. The literature data increase the heterogeneity of the overall effect size. We further generated a forest plot and found that the data in the literature were also meaningless. The 95% CI intersects the invalid line in the middle. After removing the literature, the heterogeneity was significantly reduced, indicating that the data on operation time may not be suitable for use in this study. In general, PN requires a longer operation time than RN. The length of the operation is closely related to the operator and the configuration of personnel and equipment related to the operation. In the subgroup analyses, results showed that a high degree of heterogeneity in the cancer histology subgroup (I^2^ = 75%). The findings presented here must be generalized with caution because of heterogeneity, or, in other words, it seems whether PN or RN is not so closely related to the histology classification of kidney cancer. However, people that were in different Fuhrman Grade may have different surgical effects. The specific operational situation in each country was uneven, and inevitable information bias may have emerged during the data collection. In view of this, a complete, large-sample, multi-country, multi-ethnic prospective randomized trial is essential to validate our findings.

## Limitations

The shortcomings of this study are as follows: 1) The original documents included after the screening were all retrospective studies that had a large risk of information bias, and failing to include prospective randomized controlled studies may have had an adverse impact on our results; 2) for some articles that did not directly provide relevant data, we used the calculation methods provided by evidence-based medicine and statistical references, which may have led to selection bias; 3) due to insufficient data, we failed to conduct a hierarchical analysis and exploration based on the classification of postoperative complications, surgical methods (open, hand-assisted, and robot-assisted laparoscopy strategies), tumor pathological classification, and ischemia-reperfusion time for further recognition of the overall effect of PN vs. RN; 4) the choice of PN or RN was determined by the surgeon, and the time span of patients included in the study was large, during which, some surgical indications in these guidelines may have changed; and 5) the experience of the surgeon is different from the level of surgery, which may have an impact on the evaluation of PN and RN.

## Conclusion

With the rise of PN as a method for the treatment of larger renal tumors, although PN is inferior to RN in terms of the operation time, intraoperative blood loss, and postoperative complications, it is still effective for larger kidney tumors. It is safe and feasible because of its outstanding preservation of kidney function, better OS, and lower all-cause mortality. PN is the first choice for the treatment of larger renal tumors; however, a more comprehensive consideration is necessary for patients with kidney masses at stage T2 or higher. Moreover, it is important to fully consider tumor factors and the deterioration of renal function before surgery to minimize postoperative complications, accelerate postoperative recovery, and improve quality of life, which is the end goal of treatment. Nevertheless, it is necessary to evaluate a larger sample size of PN vs. RN for the treatment of stage T2 or larger renal tumors, and further prospective randomized controlled studies will make the evaluation of efficacy more convincing.

## Data Availability Statement

The original contributions presented in the study are included in the article/supplementary material. Further inquiries can be directed to the corresponding author.

## Author Contributions

RH, CZ, XW, and HH acquired, analyzed, and interpreted the data and drafted the paper. HH conceptualized the study and design, analysis, and interpretation of data. RH performed manuscript writing and editing. All authors contributed to the article and approved the submitted version.

## Funding

This work was supported by grants from the National Natural Science Foundation of China (No. 81860128) and the Natural Science Foundation of Jiangxi Province (No. 20171BAB205016). The funders had no role in the performance of the study.

## Conflict of Interest

The authors declare that the research was conducted in the absence of any commercial or financial relationships that could be construed as a potential conflict of interest.
